# Latitudinal diversity of biting midge species within the Obsoletus group across three habitats in Europe

**DOI:** 10.1111/mve.12379

**Published:** 2019-04-29

**Authors:** T. W. R. Möhlmann, A. M. Bekendam, I. van Kemenade, U. Wennergren, G. Favia, W. Takken, C. J. M. Koenraadt

**Affiliations:** ^1^ Laboratory of Entomology Wageningen University and Research Wageningen The Netherlands; ^2^ IFM Theory and Modelling Linköping University Linköping Sweden; ^3^ School of Biosciences and Veterinary Medicine University of Camerino Camerino Italy

**Keywords:** *Culicoides*, bluetongue, Obsoletus complex, Onderstepoort light trap, livestock disease, Schmallenberg, species composition, vectors

## Abstract

*Culicoides* species from the Obsoletus group are important vectors of bluetongue and Schmallenberg virus. This group consists of several species that cannot easily be identified using morphological characteristics. Therefore, limited information is available about their distribution and habitat preferences. In this study, we aimed to elucidate the species composition of the Obsoletus group in three habitat types at climatically different latitudes across Europe. Traps were placed in three habitat types in three countries at different latitudes. After DNA extraction, biting midges were identified using PCR and gel electrophoresis. Extraction of DNA using Chelex proved to be a cost and time efficient method for species identification. A latitudinal effect on the relative abundance of species from the Obsoletus group was found. Species composition was unique for most country‐habitat combinations. The majority of biting midges were either *C. obsoletus* s.s. or *C. scoticus*, and both species were found at all latitudes and habitats. Their wide distribution and their high abundance at livestock farms make these species likely candidates for rapid farm‐to‐farm transmission of pathogens throughout Europe. Our results emphasize the need to differentiate Obsoletus group species to better understand their ecology and contribution to pathogen transmission.

## Graphical abstract

The relative abundance of species from the Obsoletus group was more defined by latitude than by habitat.
The majority of biting midges from the Obsoletus group were either *C. obsoletus* s.s. or *C. scoticus*, and both species were found at all latitudes and habitats.
Extraction of DNA from biting midges using Chelex proved to be a cost and time efficient method for species identification.

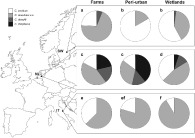

## Background

Bluetongue and Schmallenberg viruses rapidly spread throughout central, western, and even northern European countries from 2006 and 2011 onwards, respectively. Following this, it became clear that not only the biting midge *Culicoides imicola* (Kieffer, 1913) was responsible for virus transmission. While *C. imicola* was known to transmit bluetongue virus in African and south‐European countries (Venter et al. 1998; De Liberato et al. 2003), this species was not found in central or northern parts of Europe (Mellor & Wittmann 2002). Subsequently, several species of *Culicoides* were identified as potential vectors in areas outside the distribution range of *C. imicola*. These species included *C. newsteadi* Austen, 1921 (Foxi et al. 2016), *C. punctatus* Meigen 1804 (Hoffmann et al. 2009)*,* species of the Pulicaris complex (Caracappa et al. 2003), and species of the Obsoletus group (De Liberato et al. 2005; Elbers et al. 2013). Although species in the Obsoletus group are considered the most important vectors of the aforementioned viruses in northern and central Europe, it remains unclear which species within this group is the main vector.

The term “Obsoletus group” is cladistically artificial because not all species are part of a monophyletic group. However, in *Culicoides* literature it is commonly used to indicate a collection of morphologically similar species. In Europe, the Obsoletus group consists of at least five species, namely *C. chiopterus* (Meigen, 1830), *C. dewulfi* (Goetghebuer, 1936), *C. montanus* (Shakirzjanova, 1962), *C. obsoletus* sensu stricto (s.s.) (Meigen, 1818), and *C. scoticus* (Downes & Kettle, 1952) (Harrup et al. 2015; Goffredo et al. 2016). Although identification of females of these species is possible by morphological characteristics (Nielsen & Kristensen 2011; Goffredo et al. 2016), it remains a challenge even for biting midge taxonomists. This is especially the case for three species that together form the Obsoletus complex; *C. obsoletus* s.s., *C. scoticus* and *C. montanus* (Harrup et al. 2015). Because *C. montanus* has only been found in the most southern parts of Europe and not in the area under study in this paper (Goffredo et al. 2016), we refer to the Obsoletus complex as *C. obsoletus* s.s. and *C. scoticus* throughout this paper. These two species are nearly impossible to separate based on morphological characteristics. Identification of the species within the Obsoletus group and complex is therefore more reliable with molecular tools (Lehmann et al. 2012). Unfortunately, identification of species in the Obsoletus group is not consistently performed when studying *Culicoides* vectors in the field (De Liberato et al. 2005; Foxi et al. 2016). This makes it difficult to compare outcomes of different studies and, at times of disease outbreaks, to understand which species within the group are most responsible for pathogen transmission.

Species of the Obsoletus group are frequently associated with livestock, and therefore often found in large numbers at livestock farms (Elbers & Meiswinkel 2015; Steinke et al. 2016; Möhlmann et al. 2018). However, each species of the Obsoletus group seems to have its own breeding habitat and/or host preference. While *C. chiopterus* and *C. dewulfi* prefer cow dung as larval habitat, species of the Obsoletus complex (*C. obsoletus* s.s. and *C. scoticus*) have been found breeding in a much wider range of substrates (Steinke et al. 2016). Adult females of all species in the Obsoletus group seem to prefer larger livestock animals as host, although blood meals from birds and smaller mammals can also be taken (Lassen et al. 2011; Lassen et al. 2012). Viennet et al. (2013) reported that *C. obsoletus* s.s. was found to have a broader host range, and to readily bite humans and birds. When female biting midges take a blood meal from either cattle, sheep or horses, females of *C. chiopterus* favour biting on the legs, whereas members of the Obsoletus complex (*C. obsoletus* s.s., *C. scoticus*) and *C. dewulfi* prefer the head, back, and flanks (Elbers & Meiswinkel 2015). These ecological and behavioural differences suggest that we cannot consider these biting midge species as a homogenous group.

Differences in larval habitat and host preference, in combination with variable arbovirus infection rates among species of the Obsoletus group, emphasize the importance of knowing how these species are distributed in habitats throughout Europe. We therefore investigated their distribution and relative abundance in three habitat types (farms, peri‐urban, wetlands) in three countries (Sweden, The Netherlands, and Italy) representing three different latitudes in Europe. For this purpose, we employed a controlled study design with the same sampling effort across all locations. We expected species from the Obsoletus group to have specific ecological preferences for larval habitats, hosts, as well as climate. Therefore, we hypothesized that each habitat and country would represent a unique composition of Obsoletus group species.

## Methods

### *Collection of* Culicoides

Collection and identification of biting midges was earlier described by Möhlmann et al. (2018). In short, adult biting midges were collected using Onderstepoort Veterinary Institute (OVI) light traps with black light as attractant. Biting midges were sucked in by the downdraught fan, and collected in a small 500 mL bucket filled with 50 mL water‐soap solution. Traps were rotated among 27 locations spread over Sweden (surroundings of Linköping N58.410808, E15.621532), The Netherlands (surroundings of Wageningen N51.964795, E5.662898), and Italy (surroundings of San Benedetto del Tronto N42.949483, E13.878503), i.e. nine locations per country, and three habitat types (farms, peri‐urban, and wetlands) within each country. Three trap locations were selected for each habitat within a country. Trapping locations, their selection criteria as well as the sampling schedule were previously described (Vogels et al. 2016; Möhlmann et al. 2018). The three countries were selected for their different climate, each representing different latitudes across Europe. Farm locations were within 50 m of open dairy cattle stables, peri‐urban locations close to residential property, and wetlands had standing water in the proximity. Traps were placed in the period from July 2014 to June 2015 except for the winter months December, January and February (and March for Sweden). Monthly collections were performed for six consecutive days in each of the countries. Traps were active for 24 h and were emptied the next day. Biting midges were sorted and stored at −20°C in Eppendorf tubes containing 70% ethanol solution.

### *Selection of samples*

Female *Culicoides* biting midges were identified to species level with the use of the Interactive Identification Key for Culicoides (IIKC) (Mathieu et al. 2010). In total, 50 085 female *Culicoides* biting midges were collected, of which an estimated 44 406 (89%) belonged to the Obsoletus group (Möhlmann et al. 2018). From all available females of the Obsoletus group, a total of 628 was selected for molecular analysis (Table 1).

**Table 1:** Genetically identified females from the Obsoletus group.

	Farm	Peri‐urban	Wetland	Total
Sweden	98/99	6/9	88/100	192/208
The Netherlands	98/100	41/43	99/100	238/243
Italy	99/100	49/53	23/24	171/177
Total	295/299	96/105	210/224	601/628

Total number genetically identified females from the Obsoletus group per habitat (farm, peri‐urban, wetland) and country (Sweden, The Netherlands, Italy). Numbers on the right side of the backslash indicate the total number of individuals tested, whereas the numbers on the left side indicate those that could be positively identified after performance of the PCR.

From the total dataset of Obsoletus group female biting midges, 100 individuals per habitat for each country were randomly selected. If less than 100 Obsoletus biting midges were available, all individuals were used. For Sweden, 208 biting midges of the Obsoletus group were analysed, for The Netherlands 243 biting midges, and for Italy a total of 177 biting midges (Table 1). Samples that did not show a result after PCR were excluded from the dataset (Table 1).

### *Extraction methods*

DNA was extracted from individual biting midges with two extraction methods. First, a standard extraction method with the DNeasy® Blood & Tissue Kit (Qiagen, Germany) was used based on an earlier identification protocol of Obsoletus group species (Lehmann et al. 2012). The Animal Tissue Spin‐Column protocol of the DNeasy® Blood & Tissue Kit was followed according to the manufacturer's instructions. In short, individual biting midges were dried on filter paper, placed in a 1.5 mL tube, quickly frozen in liquid nitrogen and subsequently crushed with a pestle. The sample was lysed for one hour, and purified DNA was eluted in 50 μL low‐salt buffer. This extraction method was relatively costly and time consuming for the processing of many samples. We therefore decided to use a second extraction method based on Chelex (Miura et al. 2017), that was more cost efficient and faster than the extraction with the commercial kit. For this protocol, 30 μL of 5% Chelex® 100 resin (143–2832 BioRad) in ultrapure water was added to each sample in a 96 well plate. After adding 2 μL 0.5 mg/mL Proteinase K (Ambion, The Netherlands), the samples were incubated at 56°C for 24 h, followed by 3 min at 99.9°C in a PCR machine. Samples were subsequently centrifuged for 30 s at 4700 rpm. Extracts from both methods were stored in the freezer at −20°C before further use.

### Culicoides *identification*

For differentiation among species within the Obsoletus group the protocol as described by Lehmann et al. (2012) was used. Ingredients for the mastermix were adjusted for materials generally used for PCR in our laboratory. For amplification of the cytochrome c oxidase subunit I (COI) region, reverse primer PanCuli‐COX1‐727R (5′‐TATAAACTTCDGGRTGNCCAAARAATC‐3′) and species specific forward primers: *C. dewulfi* dew‐COI‐fwd (5′‐CGCCCGACATAGCATTCCCT‐3′), *C. obsoletus* s.s. obs‐COI‐fwd (5'‐CAGGAGCTTCTGTAGATTTGGCT‐3′), *C. scoticus* sco‐COI‐fwd (5′‐CCACAATTATTAATATGCGATCTACC‐3′), and *C. chiopterus* chio‐COI‐fwd (5'‐CCTTTATTTGTTTGGTCTGTTCTTC‐3′) were used. The mastermix for one sample consisted of 5 μL of 5X colourless reaction buffer, 6 μL of MgCl_2_ (3 mM), 5 μL dNTPs (1 mM), 2 μL of the forward primer (10 μM), 0.5 μL of every reverse primer (10 μM), 0.125 μL GoTaq polymerase (5 U/μL) (Promega, United States), 3.375 μL MilliQ, and 3 μL target DNA obtained from DNA extraction. The total volume of 25 μL was used for amplification with PCR settings on 15 min at 94°C, followed by 42 cycles of 30 s at 94°C, 45 s at 63°C, 45 s at 72°C, and a final step of 5 min at 72°C. Final temperature was kept at 4°C until samples were stored in the freezer at −20°C before further use.

PCR products (10 μL) were mixed with Orange G loading dye (5 μL) and loaded on a 1.5% agarose gel for electrophoresis for 45 min at 80 V. A 100 bp ladder was used as reference, as well as a negative control and positive controls for each of the four species. After electrophoresis, the gel was exposed to UV light in a Bio‐Rad Gel Doc and imported into computer program Quintify One to visualise the bands. Species were identified according to differences in PCR product length whereby *C. dewulfi* was 468 bp, *C. obsoletus* s.s. 318 bp, *C. scoticus* 237 bp, and *C. chiopterus* 190 bp (Lehmann et al. 2012).

The first PCR products from the expected four species of the Obsoletus group were excised from the agarose gel. They were subsequently recovered by the QIAquick Gel Extraction Kit (Qiagen), and sent to Eurofins Genomics (Ebersberg, Germany) for sequencing. The received COI sequences were assembled with Geneious and tested with the use of Nucleotide BLAST against the NCBI GenBank database (https://blast.ncbi.nlm.nih.gov). All COI samples were confirmed with at least 99% identity. The DNA extracts of these samples were diluted 1:1 and used as a positive control for the rest of the PCR identifications.

### *Statistical analyses*

Main effects of country and habitat, and their within‐effects (habitats within each country, and country within each habitat) on the ratios of *Culicoides* species within the Obsoletus group were tested with Fisher‐Freeman‐Halton test using the Chisq_test function with 9999 permutations in the COIN R‐package version 1.1–3 (Hothorn et al. 2008). Significant effects (α < 0.05) were further evaluated with pairwise comparisons and corrected with the Bonferroni correction. All data were analysed in the statistical software program R version 3.2.3. (R, 2015).

## Results

Of the 628 selected biting midges from the Obsoletus group, 42 (6.7%) were identified as *C. chiopterus*, 43 (6.8%) as *C. dewulfi*, 327 (52.1%) as *C. obsoletus* s.s., and 189 (30.1%) as *C. scoticus*. The remaining 27 (4.3%) biting midges did not yield a PCR product (S1 Dataset).

**Figure 1:**
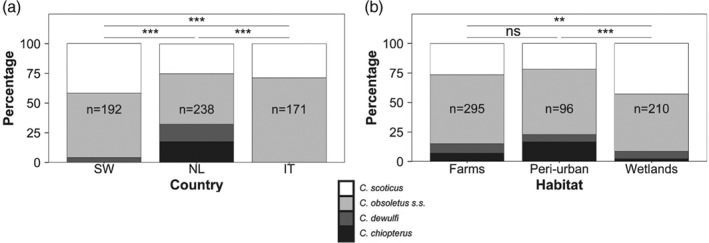
Main effects of (A) country and (B) habitat on the ratio of species in the Obsoletus group. The total sample size (n) is indicated for each bar. Significance is displayed for each pairwise comparison, with ns = not significant, ** = *P* < 0.01, *** = *P* < 0.001, SW, Sweden, NL, The Netherlands, and IT, Italy.

The ratios of species were significantly different among the three countries (χ^2^ = 122.69, df = 6, *P* < 0.001; Fig. 1A), and the three habitat types (χ^2^ = 36.03, df = 6, *P* < 0.001; Fig. 1B). Pairwise comparisons between countries showed that ratios of species were different for each comparison of the areas in Italy, The Netherlands, and Sweden (all pairwise comparisons: *P* < 0.001). In Sweden, we found three species of the Obsoletus group (*C. dewulfi*, *C. obsoletus* s.s., and *C. scoticus*), compared to four in The Netherlands, and only the two species of the Obsoletus complex (*C. obsoletus* s.s. and *C. scoticus*) in Italy. Wetlands had relatively more Obsoletus complex biting midges, and less *C. chiopterus* and *C. dewulfi*, when compared to both farms (*P* < 0.01) and peri‐urban habitats (*P* < 0.001). There was no significant difference in ratios between farms and peri‐urban habitats (*P* = 0.18).

**Figure 2:**
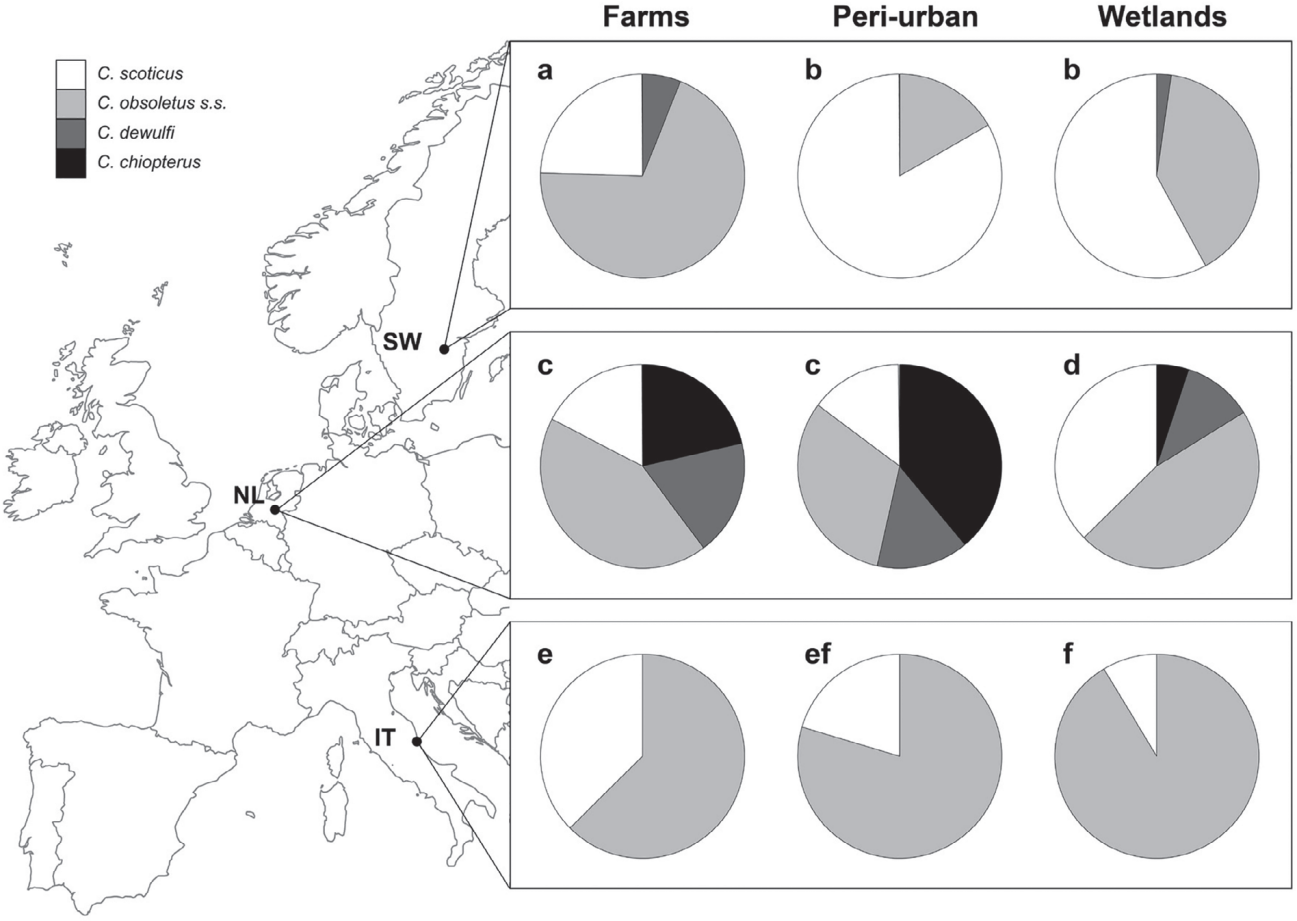
Within‐effect of habitat in each of the three countries (rows) on the ratio of the four species in the Obsoletus group, and within‐effect of country in each habitat type (columns). The sample size for each pie chart ranges from 6 to 99 (see also Table 1). Letters display significant differences among ratios shown in rows and columns at a significance level of *P* < 0.05. SW, Sweden, NL, The Netherlands, and IT, Italy.

To get more insight into the interaction between country and habitat and its effects on species composition, pairwise comparisons were made between the habitats within each country, and between countries within each habitat type (Fig. 2). Pairwise comparisons between habitat types within each country showed that farms in Sweden were significantly different from peri‐urban and wetland habitats (*P* < 0.05; Fig. 2), due to the relatively high proportion of *C. obsoletus* s.s. and lower proportion of *C. scoticus*. In The Netherlands, wetland habitats were different from the other two habitat types. Dutch wetlands had relatively low proportions of *C. chiopterus* and *C. dewulfi* and were therefore different from farm and peri‐urban habitats (*P* < 0.001). In Italy, only farms were different from the wetland habitat (*P* < 0.05), due to relatively high proportions of *C. scoticus* at farms and high proportions of *C. obsoletus* s.s. in wetlands (Fig. 2).

Ratios of the species were significantly different among countries within each of the three habitats (*P* < 0.001; Fig. 2). In Sweden, the proportions of *C. scoticus* in peri‐urban and wetland habitats were higher compared to The Netherlands, (*P* < 0.01) and Italy (*P* < 0.05). In The Netherlands the four Obsoletus group species had a more even distribution, differentiating this country from the other countries in all habitats (*P* < 0.01). In Italy, relatively high proportions of *C. obsoletus* s.s. were recorded in all habitats, except when compared with Swedish farms which had relatively more *C. obsoletus* s.s. biting midges than Italian farms (*P* < 0.05).

## Discussion

The aim of this study was to assess the distribution and relative abundance of biting midge species within the Obsoletus group in different habitats from northern to southern latitudes in Europe. We found a strong latitudinal effect on the relative abundance, as well as on the presence or absence of specific species of the Obsoletus group. Habitat types also influenced the ratios of species, but differences in relative abundance among habitat types were not consistent in areas at different latitudes.

Our study shows that the two species of the Obsoletus complex (*C. obsoletus* s.s. and *C. scoticus*) occur in the three selected European countries in relatively high proportions. The other two species from the Obsoletus group were not found in each of the studied countries. *Culicoides dewulfi* was found in collections from both Sweden and The Netherlands, while *C. chiopterus* was only found in collections from The Netherlands. Neither of these two species were recorded in the collections from Italy. These results are in line with previous findings in Sweden, The Netherlands, and Italy that demonstrated that the two species of the Obsoletus complex are the dominant species in the Obsoletus group across Europe (Meiswinkel et al. 2008; Nielsen et al. 2010; Goffredo et al. 2016; Cuéllar et al. 2018; Magliano et al. 2018). However, earlier research described the presence of the four species in each of the countries. In Sweden some individuals of *C. chiopterus* and *C. dewulfi* have been found, but they only made up around 1% of the collections, and numbers were found to be lower at more northern latitudes (Nielsen et al. 2010). Also in Italy, studies showed that *C. chiopterus*, *C. montanus*, and *C. dewulfi* can be occasionally caught, and these species made up a maximum of 3% of the total catches (Gomulski et al. 2005; Goffredo et al. 2016). The generally rare findings of *C. chiopterus* could be the result of biased sampling of this species in light traps (Carpenter et al. 2008). Yet our results do not support this, because at peri‐urban habitats in The Netherlands, with the use of blacklight traps, we identified *C. chiopterus* as 39% of the collected biting midges.

Similar to our findings, *C. obsoletus* s.s. was more abundant than *C. scoticus* throughout Italy (Magliano et al. 2018). Only in the most southern parts of Italy *C. scoticus* was relatively more abundant (Goffredo et al. 2016). For The Netherlands, our finding on the relative abundance of the four species is comparable to earlier work (Meiswinkel et al. 2008). It has been suggested that *C. obsoletus* s.s. and *C. scoticus* are adapted to a wider range of habitats and are more resistant to extreme temperatures (Nielsen et al. 2010). Furthermore, the availability of suitable hosts (horses, cattle, sheep, goats) for female biting midges, or that of larval breeding sites (i.e. dung, edges of ponds, marshes, tree holes) could influence the relative abundances of species found.

Our study shows that the relative abundance of species was significantly different between farms and wetlands in all three areas studied, despite the relative close proximity of the habitats within each country. Apparently, species within the Obsoletus group differ in how well they can take advantage of these local habitats. However, species composition of farm and wetland habitats was not consistent among countries. Wetlands in Sweden and The Netherlands had lower proportions of *C. obsoletus* s.s., and higher proportions of *C. scoticus* than farms, while this was the opposite for wetlands and farms in Italy. Although *C. scoticus* was found in relatively high proportions in wetlands in Sweden and The Netherlands, it should be noted that absolute numbers of collected biting midges were 5 to 1300 times higher at farms when compared to wetlands (Möhlmann et al. 2018). These high abundances of biting midges and their proximity to livestock animals make farms relatively favourable habitats for arbovirus transmission.

Our study is the first to compare relative abundances of Obsoletus group species for three habitats at different latitudes with a controlled study design and with the same sampling effort for each location. As a result, we can provide an unbiased overview of species composition at a large geographic scale. However, we also note that the size of the sub‐samples of the available Obsoletus group specimens (in which all nine unique habitat‐country combinations were represented) is limited. In one case, for example, only nine Obsoletus group specimens were collected during a 1‐year sampling effort (peri‐urban habitat in Sweden). In addition, the study was carried out in only one region per country. These points emphasize that our collections were local, and cannot easily be extrapolated to country level.

Molecular identification of many samples can be labour intensive and relatively expensive. In this study we show that alternative methods can be used. We first used the DNeasy Tissue Kit for DNA extraction, as described by Lehmann et al. (2012). We then tested a more cost efficient and high throughput method. DNA extraction with Chelex can be performed on whole biting midges or different body parts. In addition, it can be processed in 96‐well plates and with less handling steps than earlier protocols. The results were consistent, and many more samples can be processed with the same time investment.

From the 628 biting midges used for molecular identification, 27 (4.3%) did not yield a PCR product. These could represent failed DNA extractions, or they could include uncharacterized new species (Ander et al. 2013; Meiswinkel et al. 2015). These samples could also be *C. montanus*, as specific primers for this species were not included in the PCR. However, thus far *C. montanus* has only been identified in relatively low numbers in southern Italy (Goffredo et al. 2016), and we therefore did not expect to find *C. montanus* in this study.

The issue of reliable identification becomes even more relevant now that new (cryptic) species are being identified within the Obsoletus group (Gomulski et al. 2005; Ander et al. 2013; R. Meiswinkel et al. 2015). Apparently, the Obsoletus group consists of even more genetically different species than just five. As long as we do not make a distinction between the species when research is conducted, either in the field or in laboratory vector competence studies, we will not fully understand how and by which species, biting midge‐borne viruses could be transmitted.

## Conclusions

A strong country effect, indicative of latitudinal effects, on the relative abundance of species from the Obsoletus group was found. While the Obsoletus complex (*C. obsoletus* s.s., *C. scoticus*) was found at all latitudes, *C. chiopterus* was only identified in samples collected in The Netherlands, whereas *C. dewulfi* was not found among samples originating from Italy. Habitat types also influenced the ratios of species within the Obsoletus group, but effects were not consistent at different latitudes. Our suggestion to use a more efficient method for identification of Obsoletus group species may encourage others to also perform species identification, so that this becomes routine practice for *Culicoides* studies. The majority of the biting midges identified was part of the Obsoletus complex (82.2%), and both species were found at all latitudes and in all habitats. Their known susceptibility to viruses in combination with their wide distribution and high densities at livestock farms make *C. scoticus* and especially *C. obsoletus* s.s. likely candidates for rapid spread of midge‐borne viruses throughout Europe.

## Conflict of interest

The authors declare that there is no conflict of interest.

## Supporting information


**Table S1.** Dataset. Primary data of selected and analysed Obsoletus group biting midges.
